# Gaps and opportunities for measuring equity with the Translational Science Benefits Model: Recommendations from the Center for American Indian and Alaska Native Diabetes Translation Research

**DOI:** 10.1017/cts.2024.638

**Published:** 2024-10-24

**Authors:** Amy G. Huebschmann, Angela G. Brega, Sarah A. Stotz, Aliassa L. Shane, Roxanna King, Valarie Blue Bird Jernigan, Kaylee R. Clyma, R. Turner Goins, Gary L. Ferguson, Tassy Parker, Nathania Tsosie, Sara J. Mumby, Spero M. Manson, Meredith P. Fort

**Affiliations:** 1 Division of General Internal Medicine, University of Colorado School of Medicine, Center for American Indian and Alaska Native Diabetes Translation Research (CAIANDTR), Ludeman Family Center for Women’s Health Research, Adult and Child Center for Outcomes Research and Delivery Science, University of Colorado Anschutz Medical Campus, Aurora, CO, USA; 2 Centers for American Indian and Alaska Native Health, Colorado School of Public Health, University of Colorado Anschutz Medical Campus, Aurora, CO, USA; 3 Department of Food Science and Human Nutrition, Colorado State University, Fort Collins, CO, USA; 4 Southcentral Foundation, CAIANDTR Alaska Satellite Center, Anchorage, AK, USA; 5 Center for Indigenous Health Research and Policy, Oklahoma State University Center for Health Sciences, CAIANDTR Central Plains Satellite Center, Tulsa, OK, USA; 6 CAIANDTR Southeast Satellite Center, College of Health and Human Sciences, Western Carolina University, Cullowhee, NC, USA; 7 Institute for Research and Education to Advance Community Health (IREACH), Elson S Floyd College of Medicine, Washington State University, CAIANDTR Pacific Northwest Satellite Center, Pullman, WA, USA; 8 Department of Family and Community Medicine and Colleges of Population Health and Nursing, Public Health Institute for Indigenous Knowledge and Development, Center for Native American Health, Associate Vice President for American Indian Health Research & Education, CAIANDTR Southwest Satellite Center, Albuquerque, NM, USA; 9 Department of Family and Community Medicine, University of New Mexico Health Sciences, Center for Native American Health, University of New Mexico School of Medicine, CAIANDTR Southwest Satellite Center, Albuquerque, NM, USA; 10 Department of Health Systems, Management and Policy, Centers for American Indian and Alaska Native Health, Colorado School of Public Health, University of Colorado Anschutz Medical Campus, Aurora, CO, USA

**Keywords:** Implementation science, stakeholder participation (community engagement), diabetes mellitus, health impact assessments, health equity

## Abstract

Translational research needs to show value through impact on measures that matter to the public, including health and societal benefits. To this end, the Translational Science Benefits Model (TSBM) identified four categories of impact: Clinical, Community, Economic, and Policy. However, TSBM offers limited guidance on how these areas of impact relate to equity. Central to the structure of our Center for American Indian and Alaska Native Diabetes Translation Research are seven regional, independent Satellite Centers dedicated to community-engaged research. Drawing on our collective experience, we provide empirical evidence about how TSBM applies to equity-focused research that centers community partnerships and recognizes Indigenous knowledge. For this special issue – “Advancing Understanding and Use of Impact Measures in Implementation Science” – our objective is to describe and critically evaluate gaps in the fit of TSBM as an evaluation approach with sensitivity to health equity issues. Accordingly, we suggest refinements to the original TSBM Logic model to add: 1) community representation as an indicator of providing community partners “a seat at the table” across the research life cycle to generate solutions (innovations) that influence equity and to prioritize what to evaluate, and 2) assessments of the representativeness of the measured outcomes and benefits.

## Introduction

The Center for American Indian and Alaska Native Diabetes Translation Research (CAIANDTR) is one of seven Centers for Diabetes Translation Research (CDTR) funded by the National Institute of Diabetes and Digestive and Kidney Diseases (NIDDK) [1]. Our mission is to improve and expand translational research focused on preventing and treating diabetes in American Indian and Alaska Native (AI/AN) communities. To accomplish this mission, the CAIANDTR leverages partnerships with seven independent, regional Satellite Centers across the country, a collaborative structure that is unique among the NIDDK CDTRs (Appendix Figure). These Satellite Centers use community-based participatory research (CBPR) approaches to identify priorities with their local AI/AN community partners, to disseminate CAIANDTR resources related to diabetes prevention and treatment, and to develop community-engaged strategies to address the disproportionately high prevalence of diabetes in AI/AN populations (13.6% vs. 6.9% for non-Hispanic Whites) [2]. As identified in the Appendix Figure, the Satellite Centers are led by researchers of AI/AN heritage with Indigenous health research expertise and strong partnerships with their local AI/AN communities.

The CAIANDTR includes the following major activities:

1. Training and consultation for affiliated diabetes translation researchers;
2. Engagement with nonacademic partners seeking to pursue diabetes translation research for AI/ANs (e.g., Tribal Epidemiology Centers);
3. Educational programing for affiliated researchers as well as our diverse national network of health care, community, and policy partners;
4. Funding and training for investigators committed to pursuing a career consistent with the mission of the CAIANDTR.

**Figure 1.**
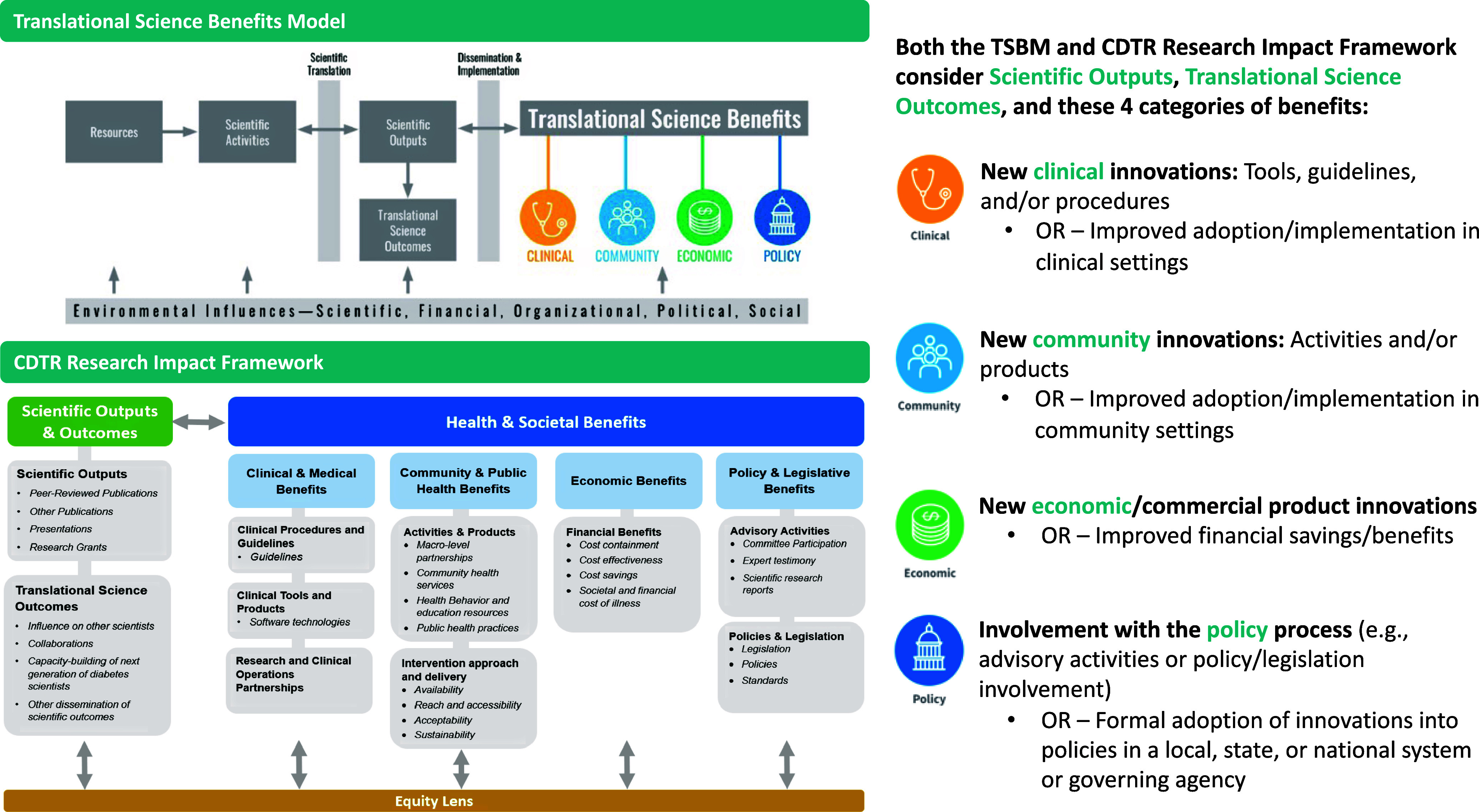
Comparison of Translational Science Benefits Model and CDTR Research Impact Framework. Abbreviations: CDTR = Centers for Diabetes Translation Research; TSBM = Translational Science Benefits Model [4]. The elements of the CDTR Research Impact Framework were developed from TSBM [5].

Given the need to justify investments in research to Congress and other agencies, there has been increasing attention on how to report the health and societal impact of research projects [3,4]. Beginning in FY2022-2023, to better demonstrate health and societal impact of the CDTRs, NIDDK recommended a new reporting tool – the CDTR Impact Framework. The CDTR Research Impact Framework is based on the Translation Science Benefits Model (TSBM) [4,5], and both frameworks encourage investigators to report on outcomes of impact beyond academic metrics of journal publications and grant funding (see Figure 1).

Both the TSBM and CDTR Research Impact Framework include four categories of impact in terms of Translational Health and Societal Benefits: a) Clinical, b) Community, c) Economic, and d) Policy. Specific examples in the “Clinical” category include new clinical benefits, such as medications/devices, or increased adoption/implementation of existing clinical benefits (see Figure 1). “Community” examples include new community health services or health behavior resources. In the “Economic” category, benefits include financial savings or revenue. “Policy” examples include advisory activities to support the development or implementation of policies to improve health/healthy behaviors at the local, regional, or national level. Of note, TSBM recommends reporting on actual benefits to date as well as potential benefits that may logically accrue but could not be measured during the life of a research effort.

**Figure 2.**
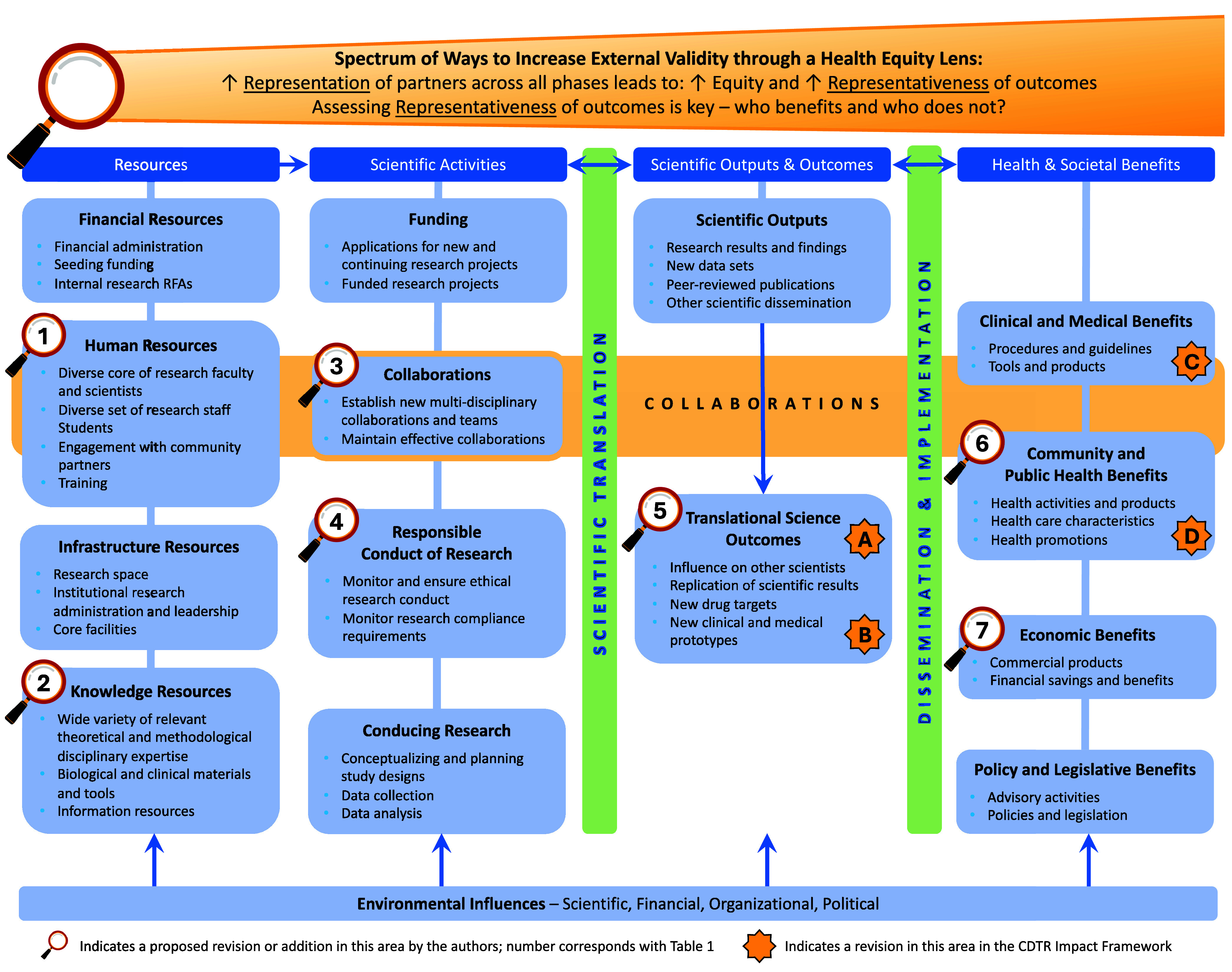
Proposed novel elements to measure within TSBM related to representation and representativeness. Abbreviations: TSBM = Translational Science Benefits Model; CDTR = Centers for Diabetes Translation Research; figure adapted from TSBM by Luke et al., 2018 [4], CDTR Research Impact Framework by Schmittdiel et al., 2024 [5], with influences from Trinkley et al., 2023 [29]. See tables 1 and 2 for descriptions of the novel elements proposed.

The creators of TSBM articulated a TSBM Logic Model (Figure 2) to further define the elements in each stage of the model, depicting three categories proximally (to the left) of the Translational Health and Societal Benefits categories of impact. These three categories are Resources (first column), Scientific Activities (second column), and Scientific Outputs & Outcomes (third column). The logic depicted is that, moving from left to right, one leverages Resources and Scientific activities to generate Scientific Outputs & Outcomes that should facilitate progress toward Translational Benefits.

**Table 1.** Proposed novel additions or revisions to the Translational Science Benefits Model (TSBM) Logic Model from the CDTR Research Impact Framework [5]

Proposed Novel Additions or Revisions to TSBM Logic Model	Symbol Designation (TSBM Domain) in Fig. 2	Related Current TSBM Logic Model Sub-domain and/or Elements	Rationale for Inclusion
***A. Collaborations***	**A** (Translational Science Outcomes)	**Sub-domain**: Collaborations **Related element**: Maintain effective collaborations	Outcomes of diverse, multisectoral partnerships that provide potential to close the gap between research, practice, and real-world impact, and that provide robust platforms for conducting health equity research.
***B. Capacity-building of the next generation of scientists***	**B** (Scientific Outputs & Outcomes)	**Sub-domain:** Translational Science Outcomes **Related element:** Influence on other scientists	Support additional researchers to conduct translational research.
***C. Clinical operations and research partnerships***	**C** (Health & Societal Benefits)	**Sub-domain**: Clinical & Medical Benefits	Support partnerships between clinical operations and research teams.
***D. Macro-level partnerships***	**D** (Health & Societal Benefits)	**Sub-domain**: Community & Public Health Benefits	Partnerships between community organizations, public health and clinical organizations, and/or the research team that seek to improve the health outcome targeted in the earlier scientific activities and/or to address other shared health priorities.

**Table 2.** Proposed novel addition or revisions to the Translational Science Benefits Model (TSBM) Logic Model to promote health equity

Proposed Novel Additions or Revisions to TSBM Logic Model	Symbol Designation (TSBM Domain) in Fig. 2	Related Current TSBM Logic Model Sub-domain and/or Elements	Rationale for Inclusion
***1a. Engagement by research teams of community partners with relevant lived experience***	**1** (Resources)	**Sub-domain**: Human Resources **Related element**: Engagement with community partners	To promote equity, it is important for research teams to engage authentically with individuals with relevant lived experience drawn from community partner organizations representing diverse cultural perspectives – such individuals provide community representation and convey key local community context (see also revision 2a). The “4 R’s” of Respect, Reciprocity, Relevance, and Responsibility should be upheld in the partnership-development process. [14,15].
***1b. Research team members with relevant lived experience***	**1** (Resources)	**Sub-domain**: Human Resources **Related elements**: Diverse core of research faculty and scientists; Diverse set of research staff	Ensuring that there is representation of research team members with lived experience is a form of power sharing and provides accountability to allow for community partner input to be incorporated into problem definition, methods, and research team plans. [9,12,40].
***2. Local community context on priorities, needs, and resources***	**2** (Resources)	**Sub-domain**: Knowledge Resources **Related element**: Information resources	The type of knowledge resource provided by individuals with relevant lived experience (see also revision 1a for details) includes: 1) current and historical community context; 2) community prioritization of important outcomes of success and which innovations to implement– including traditional solutions; 3) local/Indigenous knowledge of needs and their relation to historical and current injustices; 4) community resources and strengths that may be leveraged to support implementation.
***3a. Community representation***	**3** (Centered in Scientific Activities – but spans all phases)	**Sub-domain**: Collaborations **Related elements**: Maintain effective collaborations	Intentionally fostering community representation and engagement across the research project life cycle through phases of Exploration, Planning, Implementation, and Sustainment. This is consistent with community-engaged research principles of promoting trust and accountability by developing community-aligned solutions with mutual value to all involved. Indicators of this collaborative approach to research include: 1) authentic engagement of community members throughout the research project life cycle – including participation on research teams, when possible, to 2) build trust, and 3) share cultural perspectives, priorities, needs and resources with other implementation partners, in order to ensure meaningful benefits to all involved. See also 1a, 1b, 2a, 3b, 5a, and 6a for additional proposed novel elements that relate to Representation.
***3b. Power sharing***	**3** (Scientific Activities)	**Sub-domain**: Collaborations **Related element**: Establish new multi-disciplinary collaborations and teams	The degree of community partner power sharing is transparently specified according to commonly used spectrums of community participation [40,41] with efforts to achieve higher levels that involve, collaborate with, and defer to community partners – particularly for projects aiming to overcome inequities.
***4. Respect data sovereignty of tribal nations***	**4** (Scientific Activities)	**Sub-domain**: Responsible Conduct of Research	Indigenous tribal nations have specific data sovereignty needs and Institutional Review Board requirements that must be followed [42,43].
***5a. Assessment of engagement as a measure of community representation***	**5** (Scientific Outputs & Outcomes)	**Sub-domain**: Translational Science Outcomes	Assessment of research engagement is a key implementation outcome – it should be assessed longitudinally across the research life cycle (e.g., during Planning, Implementation, and Sustainment phases). Outcome assessments of engagement include the Research Engagement Survey Tool (REST) [11] and should be assessed longitudinally with partners.
***5b. Community capacity***	**5** (Scientific Outputs & Outcomes)	**Sub-domain**: Translational Science Outcomes	Capacity and/or readiness to conduct future intervention research and program evaluation may improve through participation in community-engaged research; capacity building and addressing infrastructure gaps should be included as activities and are important to assess.
***5c. Representativeness (equity) of Translational Science Outcomes***	**5** (Scientific Outputs & Outcomes)	**Sub-domain:** Translational Science Outcomes	Important to assess to what degree the intended end-recipients (Reach) and intended settings and staff (Adoption, Implementation, Sustainment) did or did not participate (Adoption), implement with fidelity (Implementation), or sustain the innovation (Maintenance) and why or why not – these assessments identify potential gaps in equity. The expectation is that observed inequities in who benefits in terms of these outcomes (5c) and/or TSBM Health and Societal benefits (6b) represent **unequal distribution of impact for populations and communities**.
***6a. Assessment of sustained community-research partnerships*** *(ongoing community representation and/or collaboration)*	**6** (Health & Societal Benefits)	**Sub-domain**: Community & Public Health Benefits **Related element**: Health activities and products	Ongoing engagement or collaboration between research teams and community partners in the Sustainment phase may be termed as collaborations or sustained community-research partnerships. When present, these activities demonstrate ongoing mutual benefit for community partners and research teams to work together. Assessments of partner engagement may include the Research Engagement Survey Tool (REST), [11] social network analyses, and others.
***6b. Representativeness (equity) of impact***	**6** (Health & Societal Benefits)	**Sub-domain**: Community & Public Health Benefits **Related element**: Health promotion	Assess holistically the impact of a program on a community and identify any subgroups who receive disproportionately more or less benefit. Use a Health Equity Impact Assessment tool [16,17] to assess potential benefits and unintended consequences, conduct quantitative assessment of effectiveness by subgroups (heterogeneity of effects) and qualitative assessments, and use locally tailored survey measures. **The representativeness of community priority outcomes of success should be included here and/or in revision 5c, depending on whether they are Translational Science Outcomes (5c) or Health and Societal Benefits (6b).**
***7a. Impact on unmet social needs***	**7** (Health & Societal Benefits)	**Sub-domain**: Economic Benefits	Improvement in economic and social well-being of communities in terms of social needs, such as rates of food and housing insecurity.
***7b. Compensation for community contributions***	**7** (Health & Societal Benefits)	**Sub-domain**: Economic Benefits	Compensate community partners for their contributions throughout the life cycle of all projects.

As with any new approach for reporting research benefits, retrofitting TSBM to describe the impact of our ongoing CAIANDTR efforts poses both opportunities and challenges. We have encountered a challenge in that the importance and impact of our community-engaged research relationships that we have nurtured over decades with partner agencies and tribal organizations are not emphasized enough within the TSBM Logic Model. Furthermore, our partners’ priority outcomes for addressing health equity are not always well-aligned with TSBM. For example, TSBM does not have an explicit measure of community representation across the research life cycle.

The CDTR Research Impact Framework has already proposed thoughtful amendments to the TSBM Logic Model as shown in Figure 2 and Table 1; these specifically include the following: A) collaborations as a Translational Science Outcome (in addition to being listed as a resource in the original TSBM Logic Model), B) capacity-building of the next generation of scientists as a stand-alone element related to the existing “influence on other scientists” element, and two types of collaborative partnerships within the Translational Health and Societal Benefits categories: C) clinical operations and research partnerships, and D) macro-level partnerships. Our objective in this manuscript is to use the experience of the CAIANDTR to describe and critically evaluate gaps in the fit of TSBM as an evaluation approach sensitive to health equity issues and to propose several new elements to the TSBM Logic Model (Figure 2 and Table 2). As described by other leaders in the implementation science field [6,7], limited attention to health equity [8,9] is a concern for many implementation science frameworks, not just TSBM. Thus, both TSBM and its derivative CDTR Research Impact Framework could benefit from updates to add further emphasis on key elements that contribute to health equity, such as centering community representation using CBPR methods [8,9] across the research life cycle and assessing the representativeness of outcomes [7,10]. Our hope is that these proposed amendments to enhance the measurement of representation, representativeness, and other key elements that contribute to health equity in the TSBM Logic Model will be additive to the important existing TSBM measures articulated to date.

Drawing on others’ definitions of community engagement [11], community-engaged research principles necessary to promote health equity [12,13], and the 4 R’s of Respect, Reciprocity, Relevance, and Responsibility [14,15] in the partnership-development process, we define community representation as *an indicator of providing community partners with “a seat at the table” across the research life cycle to help generate solutions (innovations) that influence equity and to prioritize what to evaluate*. As further articulated in Table 2, under element 3a, this includes 1) authentic engagement of community members throughout the research project life cycle – including participation on research teams, when possible, to 2) build trust and 3) share cultural perspectives, priorities, needs, and resources with other implementation partners, in order to ensure meaningful benefits to all involved. Drawing on established health equity and implementation science research methods [7,16,17], we define representativeness as: *an assessment of how equally the benefits and impact of a solution (innovation) are distributed – determining who benefits, who does not, and who should – among populations, communities, and implementing organizations*.

We will first outline how the CAIANDTR’s efforts in the current funding cycle (August 2021 to January 2024) mapped onto the TSBM and CDTR Research Impact Framework to show positive existing alignments and potential gaps. Next, to address the identified gaps, we propose novel elements to measure in TSBM and the CDTR Research Impact Framework to sharpen the focus on health equity. To illustrate the impetus for these proposed revisions, we describe the impact of research and programs conducted within the CAIANDTR’s regional Satellite Centers with attention to the novel revisions proposed. We conclude with future considerations to enhance the application of these frameworks.

## Methods

### Overarching contributions of the CAIANDTR captured by TSBM outcomes in the CDTR research impact framework

To consider the potential gaps between the TSBM and CDTR Research Impact Framework and the activities of the CAIANDTR, we followed a community-engaged critical appraisal approach in our established CAIANDTR community of practice. CAIANDTR leaders held a series of three discussions in monthly meetings of the CAIANDTR Outreach, Engagement, and Dissemination Committee, which includes representation from all seven Satellite Centers, and three discussions in the monthly CAIANDTR Steering Committee meetings. We took notes from each of these meetings that informed our initial recommendations. We also had two additional discussions with the CAIANDTR Outreach, Engagement and Dissemination Committee to iteratively discuss and revise our proposed recommendations for changes based on reviewer critiques of the initial manuscript. These discussions compared the priority activities and outcomes of the Satellite Centers to these frameworks and considered any gaps and areas of misalignment. Between these meetings, CAIANDTR Steering Committee coauthors iteratively proposed revisions to augment the measures of impact in the TSBM Logic Model to incorporate equity principles and iteratively developed a visual representation of the CAIANDTR Steering Committee’s connections with the Satellite Centers and their communities as a model of important elements of community representation and impact in our work. We also co-developed case examples and vignettes from multiple Satellite Centers to highlight outcomes according to our proposed revisions to the TSBM Logic Model (Figure 2). In the Results section, we report the findings of these critical appraisal discussions and the proposed revisions to the TSBM Logic Model.

## Results

### Assessment of potential gaps in TSBM and the CDTR research impact framework with regards to health equity

Using the TSBM Logic Model with amendments from the CDTR Research Impact Framework (Figure 2), impact of the CAIANDTR in the current funding cycle is most notable in the Scientific Outputs & Outcomes of: 1) contributions to capacity-building of the next generation of scientists and clinicians, 2) facilitation of new collaborations, and 3) important research publications and presentations related to diabetes among AI/AN peoples [18–21]. In addition, Satellite Centers led CBPR activities that generated impact within the Community, Clinical, Economic, and Policy categories of impact, examples of which are shown in Table 3. However, there are notable gaps in TSBM and the CDTR Research Impact Framework as applied to the CAIANDTR – we were unable to find suitable areas of impact to document two overarching constructs: 1) Representation and 2) Representativeness, as defined above.

**Table 3.** CAIANDTR examples of representation and community priority outcomes to promote equity in addition to classic translational science benefit outcomes

Name of Initiative	Example 1 – Alaska Satellite Center Community Health Learning	Example 2 – Central Plains Satellite Center Food Sovereignty Efforts [26]	Example 3 – Rocky Mountain Satellite Center “What Can I Eat?” (WCIE) Diabetes Nutrition Education for AI/AN Adults with T2D [32–34]	Example 4 – Rocky Mountain Satellite Center SNAP-Ed Nutrition Education in Native Communities	Example 5 – Southeast Satellite Center Cultural Tailoring of Advance Care Planning [44]	Example 6 – Southwest Satellite Center Indigenous Knowledge Translation
**General initiative description**	Diabetes Wellness Fair and annual symposium to educate individuals about what services are available locally to support diabetes prevention and care.	Center for Indigenous Innovation and Health Equity (CIIHE) initiative is a community-academic partnership with the goal of strengthening Indigenous food systems and practices to promote health and well-being.	Diabetes nutrition education curriculum for AI/AN Adults living with T2D that includes Indigenous strengths and values-based approaches	SNAP-Ed in Native Communities in Colorado focuses on innovative translation of SNAP-Ed guidance to provide decolonized Indigenized nutrition education to Native communities as informed by Native-led organizations.	Cultural tailoring framework with use case example: tailoring *Make Your Wishes About You* (MY WAY) – an intervention to improve advance care planning access and completion for American Indian peoples.	Indigenous knowledge translation [45] (knowing, doing) to examine inter-relatedness of diabetes and hypertension and diabetes and dementia (“Type 3” diabetes) within community, traditional healer, tribal health provider worldviews/practices and Western science ways of knowing.
**Representation – ways that communities inform and generate solutions**	A health needs assessment survey was administered to participants and included questions regarding information community members would like to hear about related to diabetes and preferences for mode of communication.	Food Sovereignty is a culturally centered movement rooted in traditional Indigenous knowledge – these efforts directly intervene upon systems-level barriers to health for Indigenous peoples, making it an important strategy for health equity.	WCIE is developed for Native people – by Native people. The idea of WCIE came from Native community members, and WCIE development, implementation, and evaluation is led by and in partnership with Native communities [32,33].	Successful ongoing negotiation with state SNAP-Ed leadership to authentically work within “allowable costs,” re-define nutrition education evaluation, and creatively support nutrition education activities not typically supported by SNAP-Ed guidance.	15-step process of cultural tailoring MY WAY in partnership with a local tribal community advisory board and a professional advisory board – this process considered the 4 core values of individual, familial, and Tribal autonomy, as well as self-determination.	Participatory strategic planning: process is grounded in consensus building, honoring existing and prior efforts, and conducting a critical co-evaluation of “what is possible” to identify a set of priorities and a plan that is realistic and achievable (promise keeping) [46].
**Outcomes prioritized by community** **(includes representativeness when appropriate)**	Approximately 40 community members (customer-owners) attended the fair to learn about healthy eating habits, how to use a glucometer, mindfulness techniques, and more wellness factors Attendees enjoyed activities, prizes, healthy snacks, and presentations from departments/ services that encompassed a holistic approach to health	↑ Physical, emotional, and spiritual health Exposure to healthy, traditional and Indigenous foods ↑ Cultural connections, family relationships, and Tribal identity Presence of community members on the land (e.g., hunting, fishing, gathering, foraging) ↑ Transmission of traditional knowledge across generations	High satisfaction with in-person and remote WCIE classes and peer-to-peer learning and support opportunities[34] Preference for Native nutrition educators Improvement in self- efficacy of healthy eating behaviors	Multiple case study evaluation of Native/Indigenous sub- award grantees centered on using qualitative data collection to center Native storytelling values as a traditional way of knowing	Feasibility of delivering MY WAY by the local tribal community Cultural acceptability, readability, and cultural prioritization of MY WAY content	Reach those at high-risk (i.e., limited trust in healthcare) (e.g., men, youth) Integrate traditional healing in healthcare Practice patient and provider co-learning Access local data to define local priorities Implement health policy across life cycle [46]
**Representativeness – Translational Science Benefits** **– Community, Clinical, Economic, and/or Policy**	Community (actual): Activities allowed collaboration with departments across the entire ANHC and was open to the public, which includes anyone affected by diabetes (i.e., caregivers, and loved ones of those with a diabetes diagnosis) Clinical (actual): Created opportunity for community members to hear about services offered at ANHC, discuss questions with clinic staff, and learn about prevention resources Economic (expected): ↓ Societal costs of illness due to decreased diabetes incidence/complications	Community (actual): Restoration of traditional foodways, relational responsibilities between people and place, and improved biodiversity Economic (expected): ↓ Societal costs of illness due to decreased diabetes incidence and complications Policy (actual): Advocacy for federal, state, and Tribal policy and practices to support efforts related to Indigenous food sovereignty	Community (actual): new activities and increased representativeness of adoption and reach of diabetes education in Native communities Economic (expected): ↓ Societal costs of illness due to decreased diabetes incidence and complications Policy (actual): Successful negotiation with American Diabetes Association to provide WCIE materials at no charge for Native communities	Community (actual): Equitable reach – delivered to all members of Native communities without specific “inclusion criteria” to decrease stigma and honor Indigenous values of inclusivity and connection between community members Economic (expected): ↓ Societal costs of illness due to decreased diabetes incidence and complications Policy (actual): Successful support from state SNAP-Ed on innovative use of this funding to reach Native communities	Community (actual): New materials, trained champions (Support Stars) and processes to deliver MY WAY with cultural tailoring Clinical (expected): ↑ Access and receipt of quality end of life care Economic (expected): ↓ End-of-life costs of illness	Community/ Clinical (expected): ↑ acceptance of disease management messages by co-delivery from traditional healer and Western science perspectives Economic (expected): Local data use to predict morbidity, disease progression and accordingly ↓ healthcare cost, ↑ prevention effort Policy (actual): Co-created and tribal leader-vetted *Tribal Health in all Policies* policy; (expected) ↑ awareness of adoption of life cycle policies esp. ↑ support for health self-efficacy and in-home monitoring and technology

*Note:* AI/AN (American Indian/Alaska Native); SNAP-Ed (Supplemental Nutrition Assistance Program Education); ANHC (Alaska Native Health Campus – Jointly Managed by and Inclusive of Southcentral Foundation and Alaska Native Tribal Health Consortium); T2D (type 2 diabetes); WCIE (What Can I Eat? Diabetes Nutrition Education for AI/AN Adults with Type 2 Diabetes).

### Recommended revisions to TSBM

To promote greater attention to health equity, the CAIANDTR proposes several adaptations to TSBM that are shown visually within the TSBM Logic Model (Figure 2) and itemized in Table 2 – these generally seek to promote community representation across the research life cycle and to ensure the assessment of representativeness of outcomes. These proposed novel elements are in line with recent publications promoting the need to center communities’ priorities, assets, and needs to further the measurement of representativeness as part of health equity impact assessments in research [12,16,17,22,23]. Related to representation, the first research priority of the NIDDK report entitled “Pathways to Health for All” is to “strengthen community engagement through partnership, power sharing, and capacity building to improve research” [12]. In terms of representativeness, health equity impact assessments highlight the need to evaluate for heterogeneity of effects among subpopulations [16,17]. In the original TSBM logic model, the engagement process with partners and information gathered from partners were characterized as “Resources,” or were designated as “collaborations” as part of the Scientific Activities (Figure 2). The CDTR Research Impact Framework also characterized community partnerships as “collaborations” within Scientific Outputs & Outcomes (A in Table 1) and as “macro-level partnerships” in the Community category of Translational Benefits (D in Table 1). We propose to more holistically consider ways to assess these relationships beyond these siloed categories and propose novel elements to assess representation that span these categories and the research life cycle, in line with CBPR and Participatory Implementation Science recommendations (Table 2) [7,9,24]. For simplicity, we articulate Community Representation in the “Scientific Activities” column (item 3a in Table 2, with reference to other key aspects of representation as items 1a, 1b, 2a, and 3b), and we depict measurements of Representation (e.g., measurements of engagement and sustained or novel collaborations) as item 5a to measure engagement (“Scientific Outputs & Outcomes”) and item 6a to measure sustained/novel collaborations in the “Translational Health and Societal Benefits.” We find these novel proposed elements to more clearly recognize the critical role of community members in jointly guiding the focus and priority of the research from the very beginning and characterize community partnerships as giving rise to our CAIANDTR research, rather than growing out of it. This includes leveraging Indigenous ways of knowing and values as they pertain to health [23,25], by promoting **representation** of community voices to elevate community and traditional ways of knowing and overcome structural racism. Some examples of these newly proposed elements are: 1) including community members (e.g., AI/AN tribal members/leaders) as investigators and implementing staff; and 2) community-led prioritization of topics and guidance on the planning, implementation, and sustainment of innovations that draw on existing community health-enhancing solutions and/or new interventions. An example of efficient community representation in AI/AN communities includes leveraging existing AI/AN community advisory boards across the research life cycle to prioritize **measuring what matters** to the community. Examples of priority outcomes that are culturally relevant to multiple socio-ecological levels in the CAIANDTR work conducted across our Satellite Centers are the individual, family, community, and land and place measures in the Conceptual Framework of Potential Health Effects of Indigenous Food Sovereignty Initiatives) [26] and elements of the social context and social determinants of health (SDOH) [27,28].

In addition, we highlight ways to assess representativeness in the TSBM Logic Model, such as the use of a Health Equity Impact Assessment that includes evaluation of representative program reach/availability to different subpopulations (e.g., race/ethnicity, sex/gender). Specifically, we propose to measure the representativeness of community partner-prioritized outcomes as Scientific Outputs & Outcomes (5c in Table 2) and/or Translational Health and Societal Benefits (6b in Table 2) [7,10], ensuring the benefits are equitably distributed across subpopulations – for example, this would encourage efforts to **promote equitable adoption by clinical/community sites and reach** to participants, not just absolute adoption or reach.

In the following paragraphs, we turn to examples from the CAIANDTR of the Resources and Representation of invested partners, as well as examples of how the impacts on the Community (Health and Well-Being), Clinical, Economic, and Policy categories might be strengthened.

### Examples of community and clinical impacts of CAIANDTR – representation and representativeness

The CAIANDTR adopts a community strengths-based perspective and makes purposeful efforts to center our work through community representation and to assess the representativeness of our outcomes [9,23]. The highly interconnected CAIANDTR structure encourages the Satellite Centers to share Indigenous and community knowledge with each other and other investigators. While the knowledge itself may be considered as part of “Human Resources” and “Knowledge Resources” in the TSBM Logic Model, it is only through active Community Representation (3a in Table 2) that the communal sharing may yield Translational Science Outcomes of “influence on other scientists” and Health and Societal Benefits in the “Community” category in terms of “community health services” and “health behavior and education resources” (Figure 1). Going further, a richer understanding of the impact of resource-sharing within the CAIANDTR may be identified with our newly recommended assessments (see Table 2) of “Community Capacity” (5b), “Representativeness” (5a and 6c) and “Impact on Unmet Social Needs” (7a). All Satellite Center efforts incorporate community representation and these include food/nutrition security efforts to increase access to healthful food through multi-level approaches, novel ways to deliver peer nutrition education for people with diabetes, local health assessments during community events, and cultural tailoring of evidence-based palliative care interventions (Table 3). In terms of representativeness, the CAIANDTR Enrichment Program webinars have reached a wide audience of clinical and community health agencies outside of typical academic networks (e.g., Indian Health Service and tribal providers, administrators, AI/AN community members).

**Figure 3.**
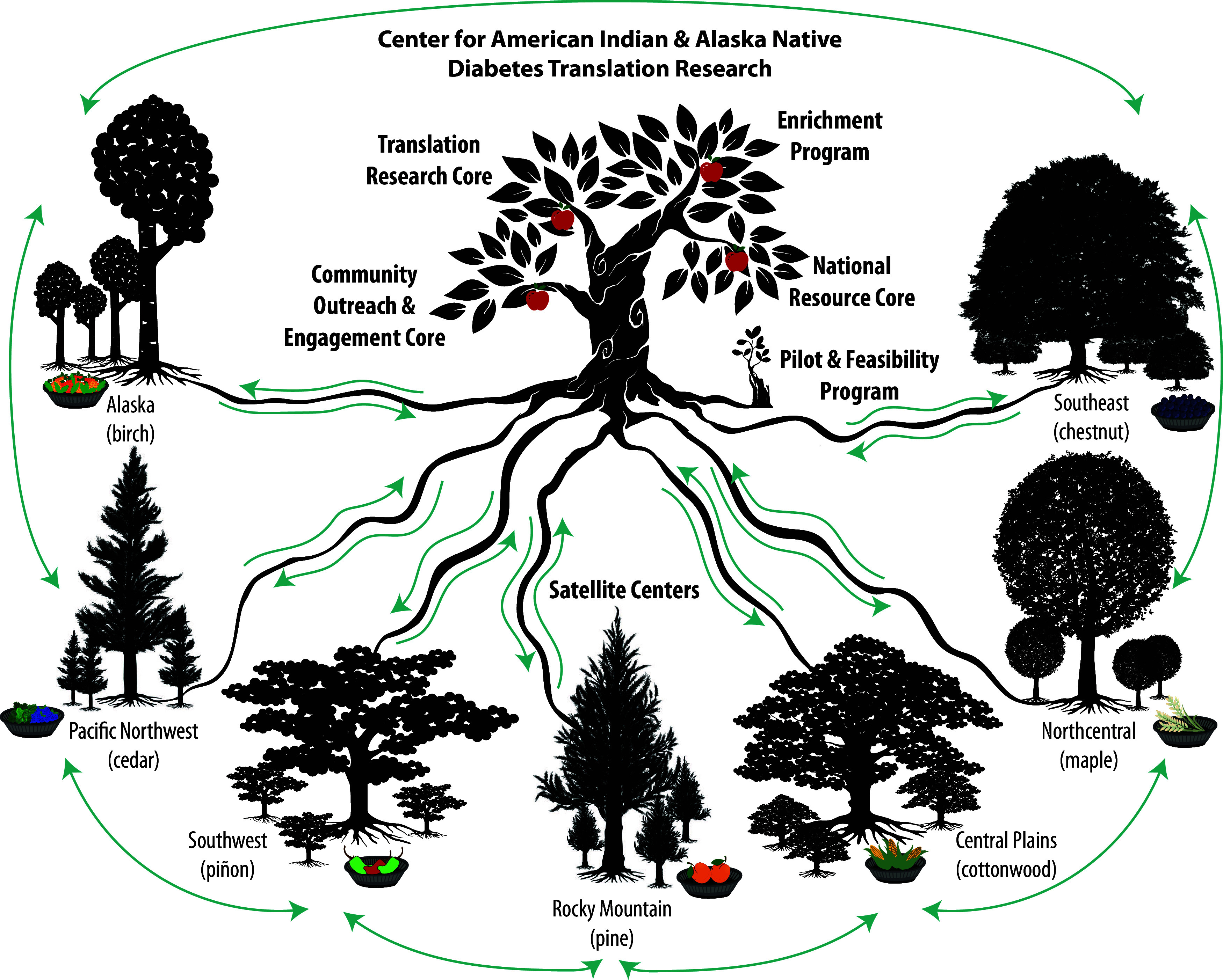
Visual model of the CAIANDTR. Each of our 7 Satellite Centers connects with their respective communities, the CAIANDTR hub, and each other. We model these multi-directional partnerships as the sharing of nutrients in a participatory and reciprocal process within the interconnected root systems of a forest ecosystem. We also model the diversity and regional connectedness of each Satellite Center visually in terms of the biodiversity of trees/plants that are local to each region (e.g., Birch, chestnut, Cottonwood, piňon trees) and their interwoven root systems locally. Depicting the fruit/produce in each region in woven baskets that are traditionally used to prepare and store produce for Indigenous peoples is a tangible illustration of the outcomes of impact according to the CDTR Research Impact Framework. The local produce depicted includes: cloudberries and spruce tips (Alaska), nettles and huckleberries (Pacific Northwest), green and red chiles (Southwest), peaches (Rocky mountain), corn (Central plains), wild rice (Northcentral), and elderberries (Southeast).

To depict how the CAIANDTR infrastructure fits with the Community Representation elements of Figure 2, we co-designed a visual representation of the dynamic interplay across the CAIANDTR as a forest (Figure 3). In the figure, each Satellite Center is represented as a tree that nurtures the AI/AN peoples in its region, as well as other Satellite Centers and the CAIANDTR hub’s Mother Tree [25,30] – this reciprocity improves the health of each Satellite Center’s community and the totality of the network through the dynamic sharing of resources and grassroots solutions from local AI/AN community networks with one another. The CAIANDTR network communicates through various mechanisms, including but not limited to conventional publications; clear and efficient community-facing infographics; newsletters and webinars that spotlight local champions and their successes for diabetes prevention/treatment; and regional strategic planning.

### Economic and policy impacts

The TSBM Logic Model allows for consideration of the “financial and societal costs of illness” related to the social drivers of health but does not explicitly highlight these elements. In terms of Economic impact, multiple Satellite Centers have worked to address healthy eating and food sovereignty by aligning tribal agricultural policies with health goals. Policy impacts have included local, tribal, and national advocacy efforts to simultaneously address healthy food production, access, and preferences to strengthen food sovereignty. This work highlights successful efforts to obtain healthy policies to advance health, including promoting food sovereignty and improving access to healthful foods in our example (see Table 3).

### Case vignettes

Two case vignette details follow for selected examples from Table 3, in order to provide additional context.

**Vignette 1** - “What Can I Eat?” Diabetes Nutrition Education for AI/AN Adults with Type 2 Diabetes (AI/AN WCIE)

*Summary:* AI/AN WCIE is a diabetes nutrition education curriculum for AI/AN adults living with type 2 diabetes (T2D) [31]. The curriculum includes Indigenous strengths and values-based approaches to diabetes nutrition education focused on traditional foods, traditional ways of knowing (e.g., storytelling), arts-based learning activities, and mindful food-related decision-making.

*Efforts to support representation and to address SDOH:* WCIE was developed by researchers in partnership with AI/AN people for AI/AN people – this includes approaches to program development, pilot evaluation, and subsequent evaluation of an added healthy food security resource. In addition, AI/AN WCIE evaluators prioritized developing a community advisory board of paid Native advisors as well as a liaison to inform AI/AN WCIE implementation and evaluation. Culturally, AI/AN WCIE includes arts-based learning activities, traditional food focus, traditional-focused mindful nutrition choice activities, storytelling and peer-to-peer interaction, and opportunities for community advocacy to promote nutrition health beyond focusing only on individuals living with T2D [32–34].

*Future opportunities:* Future steps include enhancing support from the Indian Health Service Division of Diabetes Treatment and Prevention to scale up AI/AN WCIE. Specifically, to enhance representativeness by increasing the number of AI/AN communities that have the capacity to deliver WCIE, we seek to establish support to include AI/AN WCIE as an accredited diabetes self-management education and care resource and to expand AI/AN WCIE curriculum training for community educators. Project leadership has participated in many national opportunities to speak about the program and to provide demonstrations of AI/AN WCIE to increase awareness and use of the curriculum, including a successful WCIE training in Oklahoma (August 2023) with 19 nutrition educators.

**Vignette 2** – The Center for Indigenous Innovation and Health Equity (CIIHE) Initiative

*Summary:* The CIIHE was established in 2021 by the Office of Minority Health as a community-academic partnership between Oklahoma State University Center for Health Sciences and national communities of practice – including AI/AN, Native Hawaiian, and Pacific Islander populations [35]. The CIIHE initiative developed a food sovereignty conceptual framework for health and adapted this framework to guide food sovereignty initiatives within the diverse partnering communities with a goal of strengthening Indigenous food systems and practices to promote health and well-being [26].

*Efforts to support representation and to address SDOH:* In terms of representation, the CIIHE network of community and academic partners developed the framework over the course of two years – Indigenous voices guided every stage of the process [26]. The process of developing the framework included compiling a comprehensive list of food sovereignty activities from CIIHE communities using Indigenous data collection methods, a review of the scientific literature to identify the influence of food sovereignty activities on health, including SDOH, and a talking circle with the CIIHE community partners to triangulate findings with Indigenous community guidance. In terms of representativeness, as intended, the CIIHE has been applied with several AI/AN community partners to date; the representativeness of Benefits described in Table 3 included transmission of traditional Indigenous food practices that will impact those currently involved, as well as future generations [35].

*Future opportunities:* Future steps include obtaining additional data on the relationship between health and Indigenous food sovereignty initiatives to build capacity to scale out these programs to more communities. There is interest in understanding further how engaging in food sovereignty practices improves health, in part, as a way to reconnect or support community learning about Indigenous identity and heritage.

## Discussion

### Lessons learned and future directions

Based on empirical data and examples of centering community through the CAIANDTR Satellite Centers, we proposed novel additions or revised elements to the TSBM Logic Model. These novel recommendations emphasize the importance of participatory processes that ensure community representation across the research life cycle, in order to promote representativeness of research impact in terms of TSBM outcomes and benefits. We specifically described some examples of the types of impact observed in the Satellite Centers’ work in the Community (Health and Well-being), Economic, and Policy categories of Health and Societal Impact. These participatory processes of community representation across the research life cycle that may be assessed longitudinally using measures of engagement [11] are important leading indicators of equity because they ensure community solutions, perspectives, and resources are prioritized for implementation. We also highlighted the importance of measuring representativeness as part of the TSBM Logic Model, which may be done as part of a Health Equity Impact Assessment [16,17] to determine who benefits and who does not. In the examples we provided from the Satellite Centers, community representation promoted broad and equitable reach and adoption, by working with tribal leaders and community partners to promote inclusion across the program development, roll-out, and evaluation phases.

Our proposed adaptations align with calls from other scholars in the fields of health equity and implementation science for a more specific focus of implementation science frameworks on (1) SDOH and societal context; (2) consideration of culturally relevant factors/cultural tailoring; (3) attention to potential bias/stigma in the interactions between those delivering and receiving interventions; (4) leveraging community strengths-based approaches to health promotion, and (5) assessing for inequities in outcomes among subpopulations [7,12,22,23,36]. There are limitations to our proposed recommendations, including that they are more applicable to translational research of efficacious interventions to health systems (T2–T3) and from health systems to populations (T3–T4), as compared to early-phase translational research from to develop efficacious interventions (T1–T2). Also, more formal approaches for communities of practice to propose refinements to processes are emerging that would have strengthened our critical appraisal approach – we encourage others seeking to adapt our approach to consider these methods [37].

Adding to our specific recommendations for adaptations to TSBM and the CDTR Research Impact Framework above, it may be useful for researchers and program implementers/evaluators to consider the following approaches to demonstrate impact with attention to health equity:

1. To center activities in community, researchers and program evaluators should use CBPR cycles of problem definition, assessment, interpretation, and dissemination [9]. Indicators of these participatory engagement processes should be reported as a key equity outcome of community representation in their own right as outlined in Table 2 (revisions 1a/1b, 2, 3a/3b, 5a), and these processes should be continued across the project life cycle.
2. To guide researchers who must report impacts according to the original TSBM Logic Model’s benefits to Health and Societal Impact, the TSBM online toolkit [38] may be used to give specific attention to its designated categories of impact. We encourage use of CBPR cycles to identify which outcomes matter most to community partners, as well as what potential impacts are logical to expect. NIH institutes that require researchers to report impacts according to TSBM as part of their deliverables may offer this guidance to help centers plan accordingly.
3. The Community and Clinical impacts of TSBM may be considered as part of a holistic “Health and Well-Being” category of outcomes and may consider aspects of the Indigenous determinants of health recommendations adopted by the United Nations Social and Economic Council [23,39].
4. The Economic category of TSBM could be updated to count impact on SDOH as part of the economic financial and societal costs of illness. Efforts to address these elements may also be considered as solutions in the Clinical, Community, or Policy dimensions.
5. The Policy category should include a broad lens. As currently operationalized in TSBM, this includes advocacy, such as negotiation, testimony, or writing evidence briefs to support policy change. In addition, policy change is important at multiple levels, including the tribal nation, state, and federal levels. For example, policies to advance health at a tribal level by promoting food self-determination and sovereignty are important outcomes to report. Based on the relevance of policy outcomes to advance health, the CAIANDTR is considering a policy core for the next round of NIDDK funding.

## Conclusion

We met our objective to describe potential gaps in the fit of TSBM as an evaluation approach with sensitivity to health equity issues based on the CAIANDTR experience. To fill these gaps, we proposed adding measures of community representation across the research life cycle, assessment of priority community outcomes, and the representativeness (equity) of outcomes. These proposed adaptations are fully in line with the goal of TSBM to assess impact on health and societal benefits that matter to the public and add further specificity to the “Equity Lens” that is already a part of the CDTR Research Impact Framework. These recommendations from the CAIANDTR experience are also in line with recommendations from other national leaders to add a stronger equity focus to both implementation science and diabetes translation research [6,7,12,22,23].

## Supporting information

Huebschmann et al. supplementary material Huebschmann et al. supplementary material

## Supplementary material

The supplementary material for this article can be found at https://doi.org/10.1017/cts.2024.638.
