# Supplementary figures and images for: Gaps and opportunities for measuring equity with the Translational Science Benefits Model: Recommendations from the Center for American Indian and Alaska Native Diabetes Translation Research
Source: J Clin Transl Sci. 2024 Oct 24;8(1):e206. doi: 10.1017/cts.2024.638 (PMC11626608; doi:10.1017/cts.2024.638)

# Appendix Figure – Regional Distribution of CAIANDTR Satellite Centers

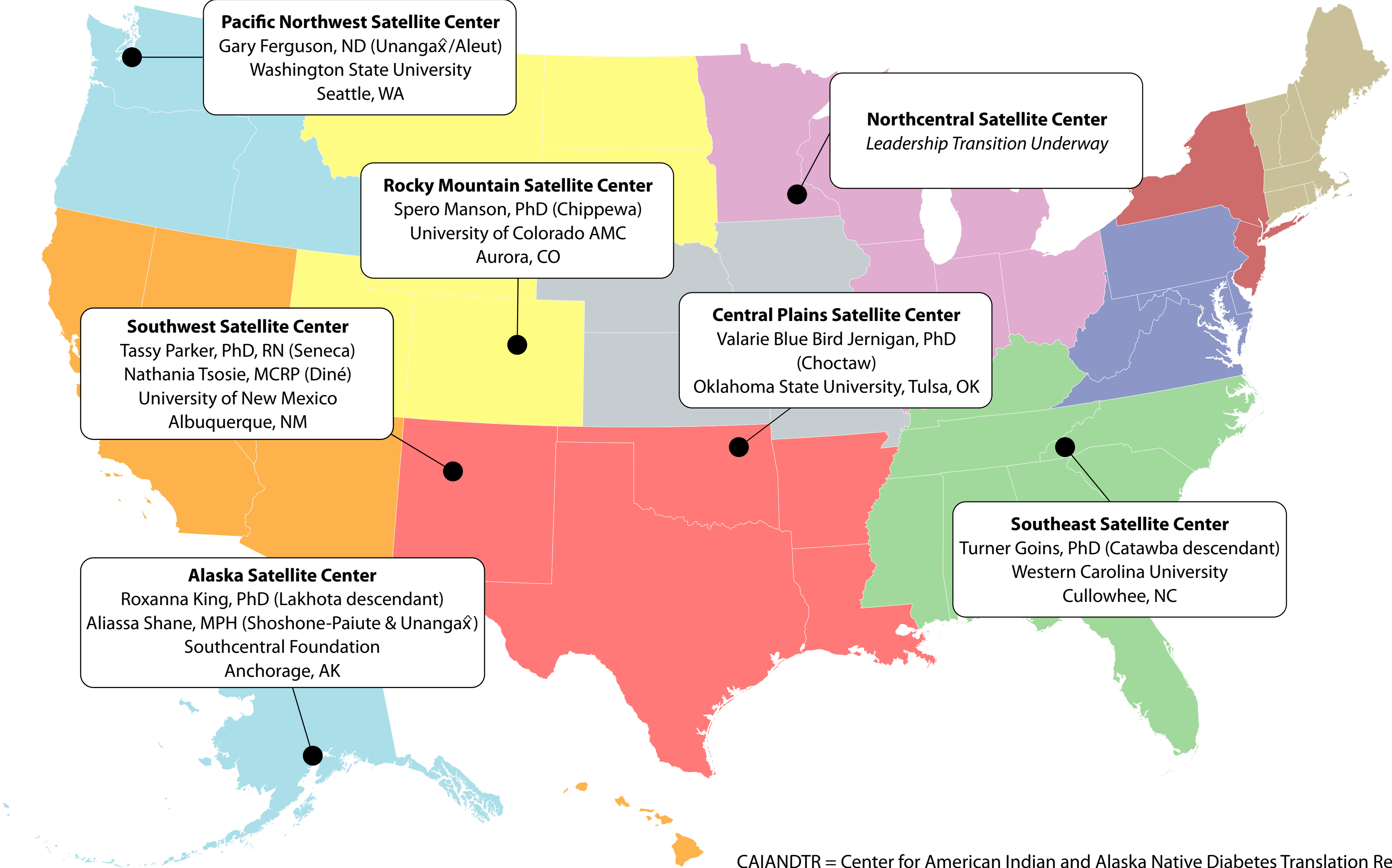

Supplement: Huebschmann et al. supplementary material [file S2059866124006381sup001.pdf]
